# Preeclampsia history and postpartum risk of cerebrovascular disease and cognitive impairment: Potential mechanisms

**DOI:** 10.3389/fphys.2023.1141002

**Published:** 2023-03-31

**Authors:** Ashtin G. Beckett, Mia D. McFadden, Junie P. Warrington

**Affiliations:** ^1^ Department of Obstetrics and Gynecology, University of Mississippi Medical Center, Jackson, MS, United States; ^2^ School of Medicine, University of Mississippi Medical Center, Jackson, MS, United States; ^3^ Department of Neurology, University of Mississippi Medical Center, Jackson, MS, United States

**Keywords:** preeclampsia, stroke, white matter lesions, postpartum, vascular dementia, Alzheimer’s disease, blood-brain barrier

## Abstract

Hypertensive disorders of pregnancy such as preeclampsia, eclampsia, superimposed preeclampsia, and gestational hypertension are major causes of fetal and maternal morbidity and mortality. Women with a history of hypertensive pregnancy disorders have increased risk of stroke and cognitive impairments later in life. Moreover, women with a history of preeclampsia have increased risk of mortality from diseases including stroke, Alzheimer’s disease, and cardiovascular disease. The underlying pathophysiological mechanisms are currently not fully known. Here, we present clinical, epidemiological, and preclinical studies focused on evaluating the long-term cerebrovascular and cognitive dysfunction that affect women with a history of hypertensive pregnancy disorders and discuss potential underlying pathophysiological mechanisms.

## 1 Introduction

The diagnosis of preeclampsia, a hypertensive disorder of pregnancy, has increased over 25% in the last 20 years (Ananth et al., 2013). Preeclampsia is characterized by new-onset hypertension presenting after the 20th week of gestation, with one or more of the following symptoms: proteinuria, low platelet count, kidney or liver abnormalities, or cerebral or visual symptoms (Gynecologists and Pregnancy, 2013). Based on the diagnosis criteria, one can appreciate that preeclampsia is a multi-organ disorder of pregnancy, affecting organs such as the lungs, kidneys, liver, and brain. Preeclampsia is the leading cause of fetal and maternal morbidity and mortality and affects 3%–8% of pregnancies (Tranquilli et al., 2012) and higher percentage of African American pregnancies. Preeclampsia can quickly progress to eclampsia, characterized by new-onset seizures or unexplained coma during pregnancy or early postpartum period. The Preeclampsia Foundation reports that eclampsia is one of the top five causes of maternal and infant morbidity and mortality, responsible for 13% of all maternal deaths worldwide (Nour, 2008). Despite these statistics, low dose aspirin is the only recommended prophylactic treatment for women at high risk of developing preeclampsia (Gynecologists and Pregnancy, 2013), and early delivery of the placenta and fetus still remains the primary course of intervention in these patients (Sibai et al., 2005; Sibai, 2006). Importantly, while the vast majority of preeclampsia cases occur in the antepartum period, preeclampsia can be diagnosed in the postpartum period as well. Magnesium sulfate has shown efficacy in preventing seizures in women with preeclampsia with severe features; however, the effect of magnesium sulfate on other preeclampsia features such as increased anti-angiogenic factors or blood pressure remains mixed (Euser and Cipolla, 2009). Thus, there remains an urgent need for the development of novel therapeutic options for preeclampsia and eclampsia.

### 1.1 Preeclampsia is associated with neurological symptoms during pregnancy

Cerebral and visual symptoms are included as potential diagnosis criteria if presenting along with new-onset hypertension in pregnancy. Cerebrovascular complications are common findings in preeclampsia/eclampsia [(pre)eclampsia] patients. Indeed, of all preeclampsia-related deaths, cerebrovascular events are the main cause in about 40% of cases, with stroke and edema being most prevalent (MacKay et al., 2001). During pregnancy, preeclampsia patients are four times more likely to have a stroke compared to normotensive patients (James et al., 2005) with hemorrhagic stroke being more common than ischemic strokes. These disproportional findings have been reported in small studies in France (Sharshar et al., 1995) and Taiwan (Jeng et al., 2004). Importantly, preeclampsia is one of the most common risk factors for antepartum and postpartum stroke (Lanska and Kryscio, 1998; James et al., 2005). The underlying pathophysiological mechanisms contributing to stroke and other cerebrovascular complications during or after (pre)eclampsia-complicated pregnancies have not been fully elucidated. Nevertheless, some of the cerebrovascular changes and potential underlying mechanisms in hypertensive disorders of pregnancy have been covered in a review paper from our group (Jones-Muhammad and Warrington, 2019). In the current review, we focus on cerebrovascular and cognitive changes that occur in the early and late postpartum periods in women with a history of (pre)eclampsia and other hypertensive disorders of pregnancy, and discuss some potential mechanisms from clinical and preclinical studies.

### 1.2 A history of (pre)eclampsia is associated with increased incidence of stroke

Women with a history of preeclampsia are at higher risk of having a stroke during the non-pregnant, postpartum period, than women with a history of normotensive pregnancies (Tranquilli et al., 2012), and a history of (pre)eclampsia is associated with a two-fold increase in the likelihood of stroke later in life (Wu et al., 2017). Additionally, a 4-5 fold increased risk of stroke has been reported for women with a history of (pre)eclampsia compared to normotensive pregnant women (Hammer and Cipolla, 2015). Of note, hemorrhagic strokes are the most frequent type of stroke affecting (pre)eclampsia patients. In a small study of 27 preeclampsia patients, 25 had hemorrhagic stroke while only two had ischemic strokes (Martin et al., 2005). This increased risk of hemorrhagic stroke has also been documented with other hypertensive disorders of pregnancy such as chronic hypertension and gestational hypertension (Bateman et al., 2006). It should be noted that not only is overall risk of stroke increased, but a history of preeclampsia is associated with an increased risk of fatal strokes as opposed to non-fatal stroke.

In addition to hemorrhagic strokes, women with a history of preeclampsia also have an increased risk of ischemic stroke. In a population study, Brown et al. (2006) showed that women with a history of preeclampsia were over 60% more likely to suffer from ischemic stroke during the postpartum period than women without a history of preeclampsia (OR: 1.63; 95% CI: 1.02–2.62 after adjustment for age, race, education, and number of pregnancies). Another study looked specifically at the incidence and causes of stroke during the peripartum and postpartum periods and showed that although the incidence of intraparenchymal hemorrhages (4.6 per 1,00,000 deliveries) was similar to ischemic stroke (4.3 per 1,00,000 deliveries), eclampsia was the leading cause of intraparenchymal hemorrhage (44%) and ischemic stroke (47%) (Sharshar et al., 1995). These findings suggest that (pre)eclampsia may increase the risk of stroke after childbirth due to hemodynamic dysfunction. In a previous review article, the relative risk for fatal stroke events after preeclampsia was found to be greater than non-fatal stroke events (RR: 2.98; 95% CI: 1.11–7.96 and RR: 1.76; 95% CI: 1.40–2.22) (Bellamy et al., 2007). Keskinkılıç et al. (2017) reported that (pre)eclampsia/Hemolysis Elevated Liver enzymes Low Platelet count (HELLP)-related intracranial hemorrhage accounted for 41.3% of hypertension-related maternal death in Turkey. Likewise, similar results were reported in a nationwide study of pregnancy-related intracerebral hemorrhage (ICH) in Japan; 26.3% (*n* = 10) of ICH in pregnant women were related to preeclampsia. The prevalence of preeclampsia-related ICH mortalities was 40% (*n* = 4) during the acute postpartum period (Yoshimatsu et al., 2014). Taken together, these studies highlight the elevated risk for later-in-life hemorrhagic and ischemic stroke in women with a history of pregnancy-related hypertensive disorders.

### 1.3 Visual changes have been reported in preeclampsia patients

Headache and visual changes are two of the most common preceding symptoms to a (pre)eclampsia episode. Multiple case studies over the past 20 years have reported visual changes in (pre)eclampsia patients during the antepartum and postpartum period (Kesler et al., 1998; Garg et al., 2013; Bereczki et al., 2016; Borovac et al., 2016). Headaches during the peripartum and postpartum period, when associated with elevated blood pressure, tend to reflect an elevation in cerebellar perfusion pressure, cerebral edema, and encephalopathy (Gestational Hypertension and Preeclampsia, 2020). Cortical blindness is an acute complication with an incidence of 1%–15% in patients with severe preeclampsia (Borovac et al., 2016). In one case study, a (pre)eclampsia patient presented with double vision at the 32nd week of gestation. On subsequent postpartum fluid-attenuated inversion recovery (FLAIR) coronal magnetic resonance imaging (MRI), vasogenic edema in the occipital cortex was found to be the main cause of the cortical blindness. The patient eventually fully recovered with no edematous lesions visible on the 6 months follow-up MRI scan (Borovac et al., 2016). Two other case studies reported transient cortical blindness presentation during the antepartum and postpartum period. In one case study, the patient’s vision gradually returned to normal after cesarean delivery was performed, but in the other case study, vision loss did not improve until a month after delivery. The cause for the vision loss in these studies was found to be vascular endothelial damage seen on MRI scans (Kesler et al., 1998; Garg et al., 2013). A prospective study performed over a 14 year period also reported cortical blindness to be a further complication of (pre)eclampsia (Cunningham et al., 1995). The data on (pre)eclampsia-induced cortical blindness demonstrate the negative changes in the structural integrity of the visual pathway leading to transient damage.

### 1.4 A history of (pre)eclampsia is associated with cognitive changes

Previous studies have shown that childbirth is associated with increased vulnerability to psychiatric episodes such as depression, anxiety, and psychosis after delivery (Postma et al., 2014; Bergink et al., 2015). Furthermore, preeclampsia has an added risk of psychiatric episodes in postpartum mothers, increasing the incidence rate ratios (IRR) from 2.93 (95% CI: 2.53–3.40) in normotensive primiparous women to 4.21 (95% CI: 2.89–6.31) in preeclampsia patients (Bergink et al., 2015). Furthermore, the highest incidence rate ratios were reported in women with a history of preeclampsia plus a somatic co-morbidity, IRR = 4.81 (95% CI: 2.72–8.50). Several studies have demonstrated higher anxiety and depression scores in preeclampsia patients several years after pregnancy (Postma et al., 2014; Fields et al., 2017). One study measured anxiety using self-report inventories on a basis of physiological symptoms such as inability to relax, rapid movement of hands, feeling of heart racing, and/or catastrophic thinking (Fields et al., 2017). Using the Beck Depression Inventory, previously normotensive pregnant women had a score of 2.0, while women with a history of preeclampsia had a score of 4.0 (Fields et al., 2017). Additionally, normotensive women had a score of 1.5, while preeclamptic women had a score of 3.0. Although the data did not reach statistical significance in either of these tests, the score was doubled for preeclampsia patients compared to normotensive patients, demonstrating a trend for higher depression and anxiety in preeclampsia patients. In another study, the Hospital Anxiety and Depression Scales were used to measure the severity of emotional disorders and reported that women with (pre)eclampsia had significantly higher scores compared to normotensive controls on both scales (Postma et al., 2014). These studies demonstrate increased susceptibility for further psychiatric care during the postpartum period in (pre)eclamptic women even decades after pregnancy when compared to their normotensive counterparts.

Cognitive impairment can range from daily absent-mindedness to errors in motor function. Very few studies have assessed the frequency of cognitive failures in postpartum women with a history of (pre)eclampsia. Two specific studies have shown a significantly higher score on cognitive failure questionnaires (CFQs) for formerly (pre)eclamptic women compared to normotensive patients (Aukes et al., 2007; Postma et al., 2014). CFQs were administered to formerly eclamptic (*n* = 30), preeclamptic (*n* = 31), and normotensive (*n* = 30) participants years after pregnancy. CFQ scores were found to be significantly higher in eclamptic women vs. normotensive controls with distractibility being the highest sub-category (Aukes et al., 2007). Similarly, Postma et al. (2014) demonstrated that women with (pre)eclampsia had a higher incidence of cognitive difficulties in their daily life than normotensive women during a long-term follow up study. Furthermore, (pre)eclamptic women scored significantly higher than normotensive women on the cognitive failure questions with forgetfulness and distractibility being the highest sub-categories in (pre)eclampsia patients. Visuomotor speed, tested using trail making test, was worse in eclamptic women than preeclamptic women, but both patient groups scored significantly worse than normotensive controls. Likewise, in another postpartum study, slower processing speed was also deemed a postpartum residual effect for hypertensive pregnancy disorders such as (pre)eclampsia, gestational hypertension, and chronic hypertension (Mielke et al., 2016). The Digit Symbol Substitution Task and Trail Making Test- Part A were used to assess processing speed. Women with hypertensive pregnancy disorders scored worse on all measures of speed processing (Digital Symbol Substitution Test mean score of hypertensive pregnancy = 41.2; normotensive pregnancy = 43.4), Trail Making Test mean seconds hypertensive (pregnancy = 45.1; normotensive pregnancy = 42.4) (Mielke et al., 2016). Furthermore, women with eclampsia or preeclampsia with pulmonary edema had lower scores on the MoCA cognitive test at the time of discharge compared to normotensive pregnant women or preeclampsia patients without severe symptoms (Bergman et al., 2021c). A recent study demonstrated that 15 years after a hypertensive pregnancy disorder (gestational hypertension or preeclampsia), significant impairments in working memory was observed in women with a history of hypertension during pregnancy (Adank et al., 2021). These studies demonstrate the increased risk for a spectrum of cognitive impairments that can affect previously (pre)eclamptic women during the postpartum period.

### 1.5 Women with a history of (pre)eclampsia have increased risk of cerebral white matter lesions

Increased cerebrovascular vulnerability after (pre)eclampsia can lead to accumulated brain damage, manifested as brain lesions and changes in brain volume. White matter lesions (WMLs) are abnormalities seen as hyperintense areas on MRI, most commonly in the elderly community (Soma-Pillay et al., 2017). The etiology of white matter lesions in relation to (pre)eclampsia is unknown, but the presence of these WMLs has been observed in many cohort studies over the past several years (Wiegman et al., 2014; Siepmann et al., 2017) (Mielke et al., 2016) (Aukes et al., 2012; Soma-Pillay et al., 2017). These WMLs have also been reported to have a causal relationship to Alzheimer’s disease as well (Abheiden et al., 2015; Soma-Pillay et al., 2017). WMLs have been observed in women with a history of hypertensive pregnancy disorders months to years after index pregnancy.

#### 1.5.1 Months postpartum

In a longitudinal study performed in South Africa, cerebral WMLs were identified and their location determined in previously preeclamptic women (*n* = 94) (Soma-Pillay et al., 2017). MRI was performed after delivery, at 6 months, and at 1 year postpartum. At delivery, 61.7% of previously preeclamptic women had identifiable WMLs, with the majority of lesions found in the frontal lobe (60%). Other locations of lesions included parietal lobe (28%) and occipital lobe (12%). At 6 months postpartum, 56.4% of previously preeclamptic women had identifiable WMLs. At 1 year postpartum, 47.9% had identifiable WMLs, with the majority of lesions (67%) located in the frontal lobe. Interestingly, the number of medications needed to control blood pressure during pregnancy was significantly associated with increased WMLs at 1 year postpartum.

#### 1.5.2 Years postpartum

The distribution and severity of WMLs years after hypertensive pregnancy (Index since pregnancy: Eclampsia: 7.6 ± 4.7 years; Preeclampsia: 5.2 ± 4.1 years; Normal pregnant: 5.0 ± 3.3 years) (Wiegman et al., 2014) were assessed, revealing that women with a history of (pre)eclampsia were more likely to have WMLs [(pre)eclampsia = 34.4%; normotensive pregnancy = 21.3%], and these lesions were more severe than in women with normotensive pregnancy history [(pre)eclampsia = 0.07 mL; normotensive pregnancy = 0.02 mL]. Majority of lesions were observed in the frontal lobe followed by parietal, insular, and temporal lobes. Other studies have reported similar results in terms of the presence and severity of WMLs years after hypertensive pregnancy disorders (Aukes et al., 2012; Wiegman et al., 2014; Mielke et al., 2016; Siepmann et al., 2017).

In addition to WMLs, changes in regional and global brain volumes have been reported in women with a history of hypertensive pregnancy disorders. One specific study reported that women with previous hypertensive pregnancies were more likely to have smaller brain volumes than those with normotensive pregnancies years after pregnancy (Mielke et al., 2016). This same study reported a trend for greater mean WML volumes in prior hypertensive women compared to normotensive women, although the data did not reach statistical significance. The prevailing hypothesis for the formation of WMLs is that they may form as a result of vasogenic edema stemming from increased blood pressure. Vasogenic edema can lead to cytotoxic edema, or cell swelling. The presence of edema is a key finding in the pathogenesis of hypertensive pregnancy disorders (Aukes et al., 2007). For this reason, white matter lesions may be found in previously hypertensive patients years after pregnancy, and can indicate residual cognitive impairments, while also increasing risk for stroke later in life (Kitt et al., 2021). In a study of the Framingham offspring assessing WMLs and hippocampal volume, WML volume increased the odds of detecting mild cognitive impairment (MCI) at baseline by 48% [OR: 1.48; 95% CI: 1.03–2.12]. Similar odds were found in the amnestic group with MCI (Bangen et al., 2017). MCI has been considered a transitional state between the normal aging process and Alzheimer’s disease (AD). Thus, these data support the hypothesis that WMLs have a causal relationship to AD through the transitional state of MCI.

In a retrospective case-control study, there was no significant difference between women with or without AD who reported previous hypertensive pregnancy disorders; however, there was a significant difference in a sub-analysis of women with early onset AD (20.4%) reporting hypertensive pregnancy disorders vs. late-onset AD (5.2%). Early-onset AD has been associated with a different pathophysiology than late-onset AD including: increased cognitive deterioration, WMLs, and higher genetic load (Abheiden et al., 2015). Other studies have shown a link between history of preeclampsia and risk of mortality from AD (Theilen et al., 2016). Other studies showed no increased risk of AD (Basit et al., 2018) or dementia (Nelander et al., 2016), although an increased risk of vascular dementia was reported in women with a history of preeclampsia (Basit et al., 2018). Furthermore, when women with a history of preeclampsia was divided based on whether they had a small for gestational age infant, the risk of vascular dementia was seven-fold higher in those with a small for gestational age infant. This relationship was not changed when AD risk was considered (Basit et al., 2018). The previous studies suggest there is evidence of long-term presence of WMLs in previously hypertensive disorders of pregnancy, and these WMLs can cause cognitive impairments that are more likely to be seen in early-onset AD patients. More long-term studies should be performed in order to determine the increased risk of residual cognitive changes due to WMLs in previously (pre)eclamptic patients.

### 1.6 Conundrum: Why antepartum changes are predominantly posterior cerebral and postpartum changes are predominantly anterior cerebral?

**FIGURE 1:**
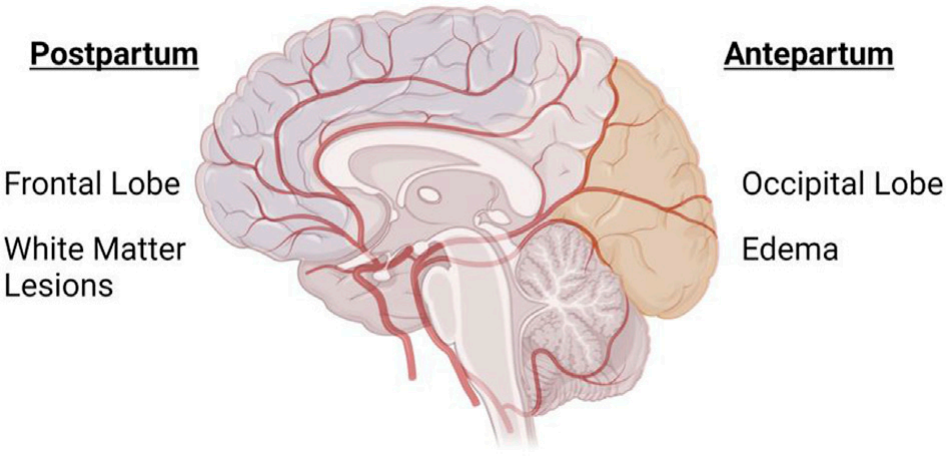
Anterior and Posterior cerebrum are differentially affected in antepartum and postpartum periods in preeclampsia. Image shows an overlay of the cerebral vasculature over the brain, highlighting the different areas of the brain that are primarily affected during (pre)eclampsia-complicated pregnancy. During pregnancy, the posterior cerebrum (occipital and parietal lobes) is most commonly affected, while white matter lesions are more prevalent in the anterior cerebrum/frontal lobe of women with a history of (pre)eclampsia. Created with BioRender.com.

In the postpartum period, white matter lesions tend to occur predominantly in the frontal lobe compared to any other brain region following (pre)eclampsia (Postma et al., 2014). During pregnancy, however, neurological findings in (pre)eclampsia patients occur mostly in the posterior cortical or subcortical region (Sharshar et al., 1995) including the parietal-occipital lobe (Topuz et al., 2008; Aygün et al., 2010; Mitas and Rogulski, 2012) (Figure 1). While not as frequent, anterior/frontal lobe abnormalities have also been reported in (pre)eclampsia patients during pregnancy (Riskin-Mashiah and Belfort, 2005). It is not known why the affected regions are different during antepartum versus postpartum periods. A histological study of postmortem vasculature revealed that posterior cerebral vessels (vertebral and basilar arteries) were structurally different from the anterior cerebral vessels, with posterior cerebral vessels having features of outward remodeling (Roth et al., 2017). While the study did not assess sex differences or directly assess the contribution of prior hypertensive pregnancy disorder, the presence of structural differences between anterior and posterior vessels is enough to hypothesize that structural differences could potentially explain the conundrum of posterior cerebral abnormalities in antepartum and anterior cerebral abnormalities in postpartum periods. It is necessary that preclinical and clinical studies consider these regional differences when designing studies.

### 1.7 Does eclampsia confer increased risk of postpartum cerebrovascular abnormalities?

The experience of seizures during pregnancy or in pregnant epileptic patients, carries increased risks. One prospective study compared dynamic cerebral autoregulation in pregnant women with eclampsia, preeclampsia with severe features, preeclampsia patients without severe features, and normotensive controls and reported decreased dynamic cerebral autoregulation in eclampsia patients [3.9(3.1–5.2)] compared to preeclampsia patients with severe features [5.6(4.4–6.8)], preeclampsia patients without severe features [6.8(5.1–7.4)], and normotensive controls [7.1(6.1–7.9)] (Bergman et al., 2022). While there have not been many studies assessing the specific risks of eclampsia to postpartum cerebrovascular injury, there is some evidence that eclampsia poses a higher risk of long-term cerebrovascular complications. For example, during pregnancy, 25/27 eclampsia patients had evidence of vasogenic edema on MRI scans; and out of 6 of these patients with infarction on imaging during pregnancy, 5 had hyperintense lesions at 6–8 weeks follow-up (Zeeman et al., 2004). Interestingly, 5 out of 6 patients with evidence of infarctions during pregnancy had experienced multiple seizures, suggesting that the severity of seizures may proportionally influence the level of damage. A case study reported an eclamptic patient who experienced vision loss, severe headache, and hemiparesis 4 days after delivery (Garg et al., 2013). While her cerebral vasoconstriction reversed, her vision did not improve up to 1 month later. Moreover, Postma et al. (2014) showed that while formerly (pre)eclampsia patients perform worse on the motor function domain of neurocognitive tasks and reported more cognitive failures than controls, there was no difference in scores between prior preeclampsia and eclampsia patients. Thus, the experience of seizures in eclampsia does not seem to impact motor function any more than preeclampsia in the postpartum years, but has worse cerebrovascular effects. Studies should be designed to assess postpartum sequelae in eclampsia patients in countries where eclampsia rates are still high.

### 1.8 Multiple pregnancies, maternal comorbidities, and onset of preeclampsia

Because a single pregnancy complicated by (pre)eclampsia increases the risk of significant neurological complications postpartum, one can speculate that having multiple pregnancies complicated by (pre)eclampsia would lead to worse outcomes for the mother. Indeed, women who had preeclampsia multiple times had an increased risk of having a stroke (Brouwers et al., 2018). Interestingly, women with two prior pregnancies affected by preeclampsia were ten times more likely to use blood pressure medication at follow-up (Magnussen et al., 2009). Studies designed to directly address this possibility are required.

Overweight [body mass index (BMI) 25–30] and obese (BMI > 30) pregnant women have a higher risk of developing preeclampsia [1.44(1.28–1.62) for overweight and 2.14(1.85–2.47) in obese] and postpartum hemorrhage [1.16(1.12–1.21) and 1.39(13.2–1.46)] (Sebire et al., 2001). Other studies have reported similar increased risk of preeclampsia, gestational hypertension, and gestational diabetes in obese and morbidly obese pregnant women (Weiss et al., 2004). Taken together, studies are needed to directly compare incidence of cerebrovascular abnormalities in women with a history of preeclampsia/eclampsia with no comorbidities versus those with preeclampsia/eclampsia and different comorbid conditions during pregnancy.

Women with early-onset preeclampsia are at a higher incidence of higher blood pressure, BMI, and abnormal lipid profiles at 9–16 years from their index pregnancy (Bokslag et al., 2017). Studies have not assessed whether early-onset preeclampsia confers greater risk of cerebrovascular abnormalities in later life compared to late-onset preeclampsia. Because early-onset preeclampsia is generally associated with severe symptoms, one could hypothesize that women who had early-onset preeclampsia would have worse cerebrovascular complications later in life compared to women with a history of late-onset preeclampsia. Studies should be designed to address this possibility.

### 1.9 Potential mechanisms contributing to increased risk of cerebrovascular impairments following (pre)eclampsia

**FIGURE 2:**
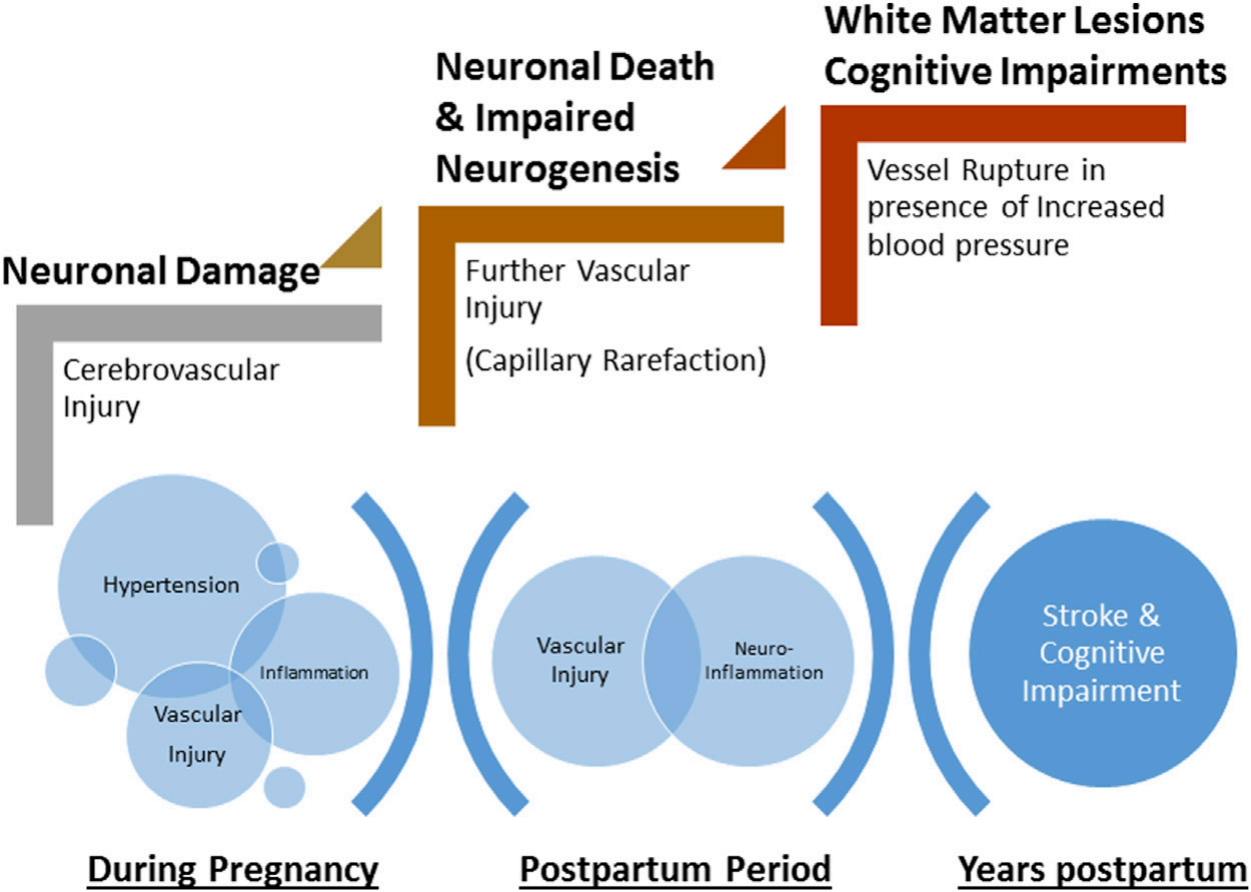
Schematic of the progressive damage to neurons and cerebrovasculature during pregnancy and the postpartum period. Hypertension during pregnancy and associated inflammation contributes to cerebrovascular injury. Incomplete repair of neurovasculature and persistent inflammation lead to further injury including capillary rarefaction and neuronal death during early postpartum period. Microscopic damage and late-life hypertension exacerbate neurovascular damage leading to rupture of vessels (stroke) and cognitive impairments.

Because preeclampsia is a hypertensive disorder, increased blood pressure, especially sudden, acute spikes, results in increased transmission of pressure to the delicate cerebral micro-vessels, leading to increased blood-brain barrier (BBB) permeability (Aygün et al., 2010; Cipolla et al., 2011). During pregnancy, (pre)eclampsia patients present with impaired cerebral blood flow autoregulation, edema, and features consistent with BBB disruption on imaging studies (Apollon et al., 2000; Demirtaş et al., 2005; Aygün et al., 2010; Mitas and Rogulski, 2012). It is possible that if vascular repair mechanisms are impaired, complete recovery of the cerebral vasculature does not occur and women enter the postpartum period with sub-clinical damage to the cerebral micro-vessels. Furthermore, if cerebral blood velocity remains elevated, there is opportunity for continuous and cumulative damage to cerebral blood vessels and neural cells. Indeed, there is evidence that this might be the case. Giannina et al. (1997) showed that at 6 and 12 weeks postpartum, cerebral and ophthalmic vessels from preeclampsia patients were still subjected to higher blood velocities compared to those from women who had normal pregnancies. This demonstrates that vessels are susceptible to pressure-induced damage in the early postpartum period. Left unrepaired, vascular injury can be exacerbated with time, contributing to long-term neurological complications (Figure 2).

Furthermore, (pre)eclampsia is considered an inflammatory disease, as affected women present with increased levels of circulating pro-inflammatory factors (Szarka et al., 2010; Cipolla et al., 2012; Pinheiro et al., 2013; Ferguson et al., 2017) and increased endothelial cell activation compared to normotensive pregnancies (Bergink et al., 2015). Endothelial cell activation increases vulnerability of the blood-brain barrier and induces functional changes to neurotransmitter metabolism and synaptic adaptability (Bergink et al., 2015). Acute disruption of the BBB was also observed in patients with posterior reversible encephalopathy syndrome in response to abrupt increases in blood pressure (Aygün et al., 2010). These pathophysiological changes can lead to changes in the structural integrity of the blood vessels and weaken the neurological circuitry. Thus, any structural damage to the cerebral micro-vessels could result in leakage of plasma constituents into the brain parenchyma, inducing neuroinflammation. Previous studies have shown that plasma from women diagnosed with preeclampsia increases the permeability of isolated cerebral veins from non-pregnant rats (Amburgey et al., 2010; Schreurs and Cipolla, 2013; Schreurs et al., 2013) and induces neuroinflammation (Cipolla et al., 2012). Taken together, leakage of plasma constituents into the cerebral parenchyma, as occurs with BBB disruption, can induce neuroinflammation and the cerebral and visual symptoms associated with (pre)eclampsia.

**FIGURE 3:**
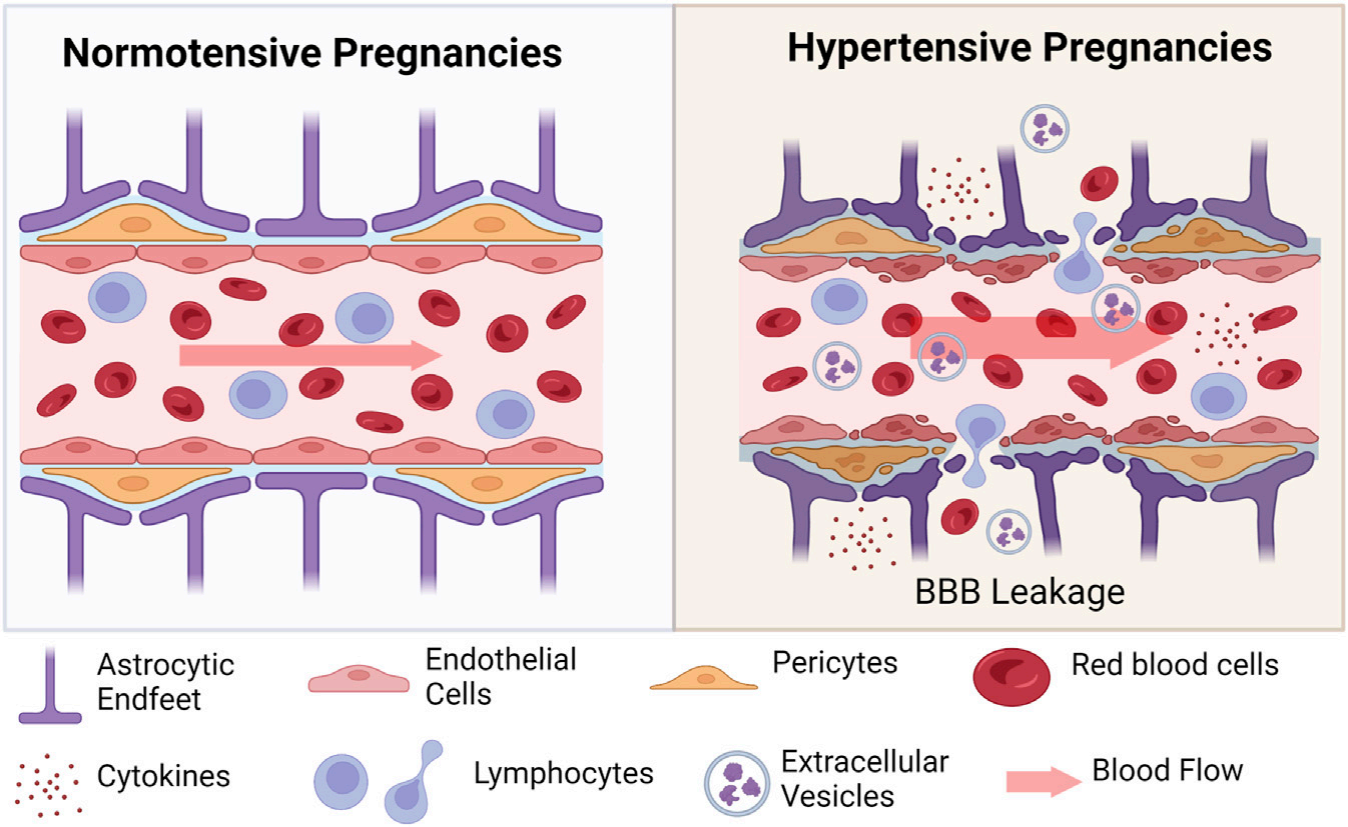
Schematic of changes at the blood-brain barrier in hypertensive pregnancies. During hypertensive pregnancies, all cells of the neurovascular unit are impacted. Endothelial cells, pericytes, and astrocytic end feet are damaged under conditions of increased blood flow (indicated by wider arrow). Damage to these cells results in extravasation of blood cells (red blood cells and various lymphocytes) into the surrounding tissue. Inflammatory cytokines and anti-angiogenic factors are also secreted into the cerebral tissues. Created with BioRender.com.

The blood-brain barrier (BBB) is formed by the close association of endothelial cells that line the blood vessels, connected *via* tight junctions. Smooth muscle cells or pericytes surround the endothelial cells and are further contacted by astrocytic end feet (Figure 3). Under physiological conditions, blood constituents such as albumin is kept within the vessels; however, following damage to the BBB, albumin and blood cells, can leak out of the vessels into the brain parenchyma, causing neuronal damage. Studies have shown that extracellular vesicles (exosomes) from preeclampsia patients can induce increased permeability in human endothelial cell culture monolayers and increase permeability to Evans blue dye in C57BL/6 non-pregnant mice (Leon et al., 2021). Furthermore, when endothelial cells were treated with plasma from preeclampsia patients, increased permeability was observed (Bergman et al., 2021a). Other studies that have assessed markers of neuronal injury or neuroinflammation in women with preeclampsia or eclampsia during pregnancy have shown increased pro-inflammatory cytokines in cerebrospinal fluid from preeclampsia and eclampsia patients compared to normal pregnant women (Bergman et al., 2021b). Increased plasma concentration of different markers of glial or neuronal damage were found in women with preeclampsia compared to normal pregnant women (Friis et al., 2022). Taken together, clinical and preclinical studies demonstrate increased BBB permeability in preeclampsia with evidence of involvement of extracellular vesicles, pro-inflammatory cytokines, and other factors during pregnancy.

In the postpartum period, circulating inflammatory factors are no longer different between women who had early preeclampsia vs. normal pregnancy at 1–3 years postpartum (van Rijn et al., 2016). Importantly, an inflammatory challenge, induced in response to influenza vaccination, resulted in an exaggerated immune response in women with a history of early preeclampsia compared to those with a normal pregnancy (van Rijn et al., 2016). Together, these studies suggest that while increased pro-inflammatory factors are no longer different in women a year after early preeclampsia diagnosis, an inflammatory challenge induces a heightened response. This observation is in line with the idea of a “second hit” phenomenon in which pregnancy is considered a stressor for women, leading to subclinical damage, exacerbated by later physiological challenges such as postpartum hypertension, obesity, diabetes, or other condition (Ferguson et al., 2017). The case for the second hit phenomenon contributing to some of the increased cerebrovascular risk factors in postpartum preeclampsia patients has been made (Ferguson et al., 2017). Thus, these later life challenges result in the manifestation of neurological disorders such as stroke, cognitive impairment, and vascular dementia.

### 1.10 Leveraging pre-clinical studies to uncover underlying mechanisms

Clinical studies have demonstrated that preeclampsia is associated with reduced utero-placental perfusion, thought to be a result of incomplete remodeling of the maternal uterine spiral arteries (Roberts and Gammill, 2005). During normal pregnancy, spiral arteries within the maternal uterus become less resistant and more compliant to allow increased blood flow to the developing placenta and fetus. This process is incomplete in preeclampsia, leading to placental and fetal hypoxia. Consequently, the ischemic placenta releases numerous factors (pro-inflammatory and anti-angiogenic) into the maternal circulation, resulting in the hallmark features of the disorder (Reviewed in (Warrington et al., 2013). To model reduced uterine perfusion, the reduced uterine perfusion pressure (RUPP) model was developed in the rat (Granger et al., 2006) and mouse (Intapad et al., 2014; Fushima et al., 2016; Jones-Muhammad et al., 2021). Preclinical studies geared at identifying postpartum cerebrovascular and cognitive changes following a (pre)eclampsia-like pregnancy are sparse. Using the rat RUPP model, persistent vascular dysfunction in isolated mesenteric arteries at 1 month and 3 months postpartum (Brennan et al., 2016) was reported. Our group later reported that at 2 months postpartum, dams subjected to the RUPP procedure during pregnancy had features of posterior cortical edema and neuroinflammation compared to sham control rats (Clayton et al., 2018). In both studies, blood pressure was no longer different between sham controls and rats subjected to RUPP in the postpartum period, demonstrating that persistent hypertension was not the underlying contributor to the vascular dysfunction observed. To our knowledge, there are no reports of cognitive function or cerebrovascular function in RUPP rats or mice in the postpartum period, although these studies are ongoing in the authors’ laboratory.

Another rodent model of preeclampsia has been used specifically for the study of superimposed preeclampsia. Superimposed preeclampsia is diagnosed in women with chronic hypertension who then go on to develop other preeclampsia symptoms after the 20th week of gestation. The pregnant Dahl salt sensitive (Dahl-SS/Jr) rat has been described as a model of spontaneous superimposed preeclampsia as it displays clinical features of preeclampsia including hypertension, proteinuria, placental hypoxia, increased sFlt-1 and TNF-alpha, and fetal growth restriction (Gillis et al., 2015). These rats also display features of disruption of cerebral endothelial of tight junctions and increased BBB permeability (Maeda et al., 2021). Our group later showed that in the postpartum period, after 2 pregnancies, Dahl-SS/Jr rats have increased pial vascular-associated microglia/macrophages compared to the normotensive Sprague Dawley rats (Warrington et al., 2022), hinting to morphological and cellular changes that are consistent with neuroinflammation.

While several pharmacological and genetic models have been described in the literature (Marshall et al., 2018), these studies focus primarily on the antepartum changes or changes in the offspring. In order to determine whether specific pathways or circulating factors contribute to the postpartum cerebrovascular sequelae, investigators ought to perform further investigations in postpartum dams after the circulating factors are removed. Using a rat model of experimental preeclampsia induced by cholesterol diet (2%) at day 7 of gestation, investigators demonstrated that 5 months postpartum, rats in the experimental preeclampsia group displayed impairments in memory and had impaired vascular reactivity to vasoactive substances (Johnson et al., 2022).

The anti-angiogenic protein, Soluble fms-like tyrosine kinase -1 (sFlt-1), has been shown to be elevated in pregnant women (Maynard et al., 2003; Rana et al., 2012). This factor sequesters the pro-angiogenic factors, vascular endothelial growth factor and placental growth factor that are important for placental vascular formation and growth during the entirety of pregnancy. Increased sFlt-1 in pregnant animals induce hypertension and fetal growth restriction and reflect pathogenesis closely associated with preeclampsia (Maynard et al., 2003; Lu et al., 2007). Moreover, reduction of sFlt-1 levels in preeclampsia patients has shown promise in delaying early delivery (Thadhani et al., 2011). While animal models of elevated sFlt-1 mimics some of the clinical characteristics, none of the studies have looked beyond pregnancy to assess postpartum neurological changes induced specifically by increased sFlt-1 during pregnancy.

## 2 Conclusion and perspectives

The studies presented in this review have highlighted the increased risk of neurological complications in women with a history of hypertensive disorders of pregnancy, specifically, (pre)eclampsia. During pregnancy, cerebral and visual symptoms are reported, and is now used as a possible diagnosis criterion if combined with new onset hypertension. In contrast to what was believed where delivery of the placenta and fetus was a “cure” for preeclampsia, emerging epidemiological and clinical studies now show increased risk for long-term neurological disorders after a pregnancy complicated by (pre)eclampsia. New research should endeavor to establish the long-term pathophysiological link between previously hypertensive pregnancy disorders and long-term residual complications. Furthermore, various interventions and therapeutics are warranted to determine whether specific pathways can be targeted to prevent the long-term neurovascular and cognitive changes in women with a history of hypertensive pregnancy disorders.
